# Psychometric properties of the unifactorial and bifactorial models of the Morisky medication adherence scale in Ecuadorians with neuropsychiatric disorders

**DOI:** 10.3389/fpsyg.2026.1764294

**Published:** 2026-04-24

**Authors:** José Alejandro Valdevila Figueira, Andrés Ramírez, Rocío Valdevila Santiesteban, Indira Dayana Carvajal Parra, Philip Morisky, María José Pico Cucalón

**Affiliations:** 1 Institute of Neurosciences of Guayaquil, Guayaquil, Ecuador; 2 Universidad Ecotec, Samborondón, Ecuador; 3 Research Network in Psychology and Psychiatry (RIPYP), Guayaquil, Ecuador; 4 Departamento de Psicología Clínica, Grupo de Investigación en Neurociencia Clínica Aplicada (GINCA), Universidad Politécnica Salesiana, Cuenca, Ecuador; 5 Casa Grande University, Guayaquil, Ecuador; 6 University of La Verne, Long Beach, CA, United States

**Keywords:** adherence, MMAS-8, neuropsychiatric illness, psychometrics, surveys and questionnaires

## Abstract

**Introduction:**

The Morisky Medication Adherence Scale (MMAS-8) is widely used to assess medication adherence, yet its psychometric properties have not been evaluated in Ecuador.

**Objective:**

This study examined the reliability, factorial structure, and convergent validity of the MMAS-8 in Ecuadorian patients diagnosed with neuropsychiatric disorders.

**Results:**

A total of 440 patients with neuropsychiatric disorders completed the MMAS-8. Internal consistency was evaluated with Cronbach’s alpha and McDonald’s omega. Confirmatory factor analysis (CFA) supported the original one-factor model with adequate fit (RMSEA = 0.08, SRMR = 0.06, CFI = 0.92, TLI = 0.90, *α* = 0.70, *ω* = 0.71). A two-factor model was also examined. Confirmatory factor analysis (CFA) supported the two-factor model with adequate fit (RMSEA = 0.08, SRMR = 0.07, CFI = 0.92, TLI = 0.90, *α* = 0.70, *ω* = 0.72). The scale showed acceptable internal consistency (ω = 0.71), and all items correlated positively (r > 0.20; *p <* 0.001). A two-factor solution produced only a slight improvement in model fit.

**Conclusion:**

The MMAS-8 appears to be a reliable and valid instrument for assessing medication adherence in Ecuadorian patients with neuropsychiatric disorders, supporting its use in clinical practice and research.

## Introduction

Adherence to pharmacological treatment is a key concept in the management of chronic and complex conditions such as neuropsychiatric disorders (NPD). The World Health Organization (WHO) defines adherence as the degree to which a person’s behavior (taking medication, following a diet, and/or making lifestyle changes) matches the recommendations agreed upon with a healthcare professional (World Health Organization, 2025.).

In NPD, adherence can be particularly challenging due to factors such as cognitive impairment, poor insight/denial of illness, adverse medication effects, complex regimens, and stigma associated with mental disorders (Organización Mundial de la Salud, 2022).

Patients with schizophrenia, bipolar disorder, major depression, epilepsy, Parkinson’s disease, or Alzheimer’s disease (among others) face significant risks derived from non-adherence, including relapses, recurrent hospitalizations, functional decline, and increased mortality (Weiden et al., 2009; Ortega Cerda et al., 2018). Therefore, accurate and systematic evaluation of adherence is essential in mental health and neurology.

A reliable assessment allows early identification of patients at risk of discontinuing treatment and facilitates individualized interventions. For this evaluation, direct methods (measurement of plasma drug levels) or indirect methods (self-reports, pill counts, pharmacy records, and structured scales) may be used (Osterberg and Blaschke, 2005).

Direct methods are often costly, invasive, and impractical for routine use. Indirect methods, although susceptible to bias, are more feasible in clinical settings. Among them, structured self-report scales are useful because they are easy to apply, inexpensive, and capture behavioral and cognitive aspects related to treatment.

In NPD, instruments must provide a multidimensional and culturally adapted evaluation, considering behavioral adherence, perceived barriers, beliefs about medication, and cognitive difficulties in following instructions.

The 8-item Morisky Medication Adherence Scale (MMAS-8) is a widely used self-report tool for assessing medication adherence. It was introduced by Morisky and colleagues, with early work reporting predictive validity; the original report was later retracted and is cited here solely for historical context (Morisky et al., 2008a). The MMAS-8 includes eight items that assess forgetfulness, voluntary discontinuation, perceived medication effect, difficulty remembering doses, and other adherence-related behaviors. Scores range from 0 to 8, classifying adherence as high (Vik et al., 2004), moderate (6–<8), or low (<6). Its design allows rapid administration and easy integration into clinical and research settings. Numerous cultural adaptations and validations support its global utility (Krousel-Wood et al., 2009).

The psychometric properties of the MMAS-8 have been studied in populations with cardiovascular diseases, diabetes, hypertension, HIV, and other chronic conditions. Findings generally show acceptable internal consistency (Cronbach’s alpha 0.65–0.83) and good construct, criterion, and discriminant validity (Vik et al., 2004; Nguyen et al., 2014).

However, the validity and interpretation of self-report adherence scales may vary across clinical populations. In psychiatric settings, adherence assessment should consider disorder-specific clinical factors. In psychotic disorders, non-adherence may be influenced by denial of illness, stigma, and low insight, which can affect the consistency of self-reported responses. In addition, the MMAS-8 has shown the need for population-specific psychometric evaluation in psychiatric outpatient settings (Staring et al., 2010; De las Cuevas and Peñate, 2015a).

Therefore, specific psychometric validation of the MMAS-8 in NPD patients is crucial, addressing semantic comprehension, factor structure, reliability, convergent validity, and predictive capacity. Although it is widely used, evidence on its psychometric performance in NPD populations remains limited, representing an important gap given the high prevalence and risks associated with non-adherence in this group. A formal validation in this population would provide a tool adapted to their clinical and cognitive needs, improving early detection of adherence problems, guiding targeted interventions, and optimizing therapeutic and functional outcomes. A validated scale also supports epidemiological studies and the design of personalized adherence programs.

In Latin America, validation and application of the MMAS-8 remain limited. Its psychometric properties have been examined in Spanish versions, including Colombian (Montesinos and Tuesta, 2022) Peruvian (Valencia Monsálvez, 2014), and Chilean samples (Berlowitz et al., 2017). These studies highlight its utility but often focus mainly on factor structure without comparing it to other adherence indicators. This narrow approach limits the understanding of construct validity, which requires demonstrating associations with additional adherence measures.

No study has yet evaluated the psychometric properties of the MMAS-8 in Ecuador, despite the considerable public health burden of NPD especially schizophrenia in young people and Parkinson’s disease in older adults. This study addresses this gap by examining the reliability and factor structure of the MMAS-8 in an Ecuadorian sample, providing information that supports future research on medication adherence in this context.

Medication adherence measurement is also increasingly relevant for interpreting patient-centered outcomes and value-based care. In outpatient settings, Morisky and colleagues showed that a brief self-report adherence measure has predictive validity for clinically meaningful outcomes, supporting its use as a practical indicator of medication-taking behavior (Morisky et al., 1986). Likewise, large trials in chronic disease have highlighted endpoints beyond physiological targets, including patient-reported outcomes and the cost-effectiveness of more intensive treatment strategies, underscoring the need to understand how real-world implementation may influence perceived benefits and health-system value (Morisky et al., 1986; Appelbaum et al., 2018). In this context, incorporating a validated MMAS-8 for Ecuadorian patients with neuropsychiatric disorders could strengthen both clinical decision-making and research by linking adherence profiles to patient-reported, functional, and resource-related outcomes in routine care (Morisky et al., 1986; Morisky et al., 2008b; Appelbaum et al., 2018).

Validation of this scale in patients with neuropsychiatric disorders is necessary because this poulation presents clinical and psychosocial characteristics that can substantially modify the construct of adherence and its measurement. Unlike other populations, these patients may experience cognitive impairments (memory, executive functions), fluctuations in reality testing, low insight into their illness, stigmatization, and complex therapeutic regimens that include polypharmacy and combination therapies. These factors not only influence adherence behavior but also how individuals understand and interpret the items on a scale. Consequently, assuming that the psychometric properties demonstrated in general populations or those with chronic non-psychiatric illnesses remain invariant in this group could compromise the validity and precision of the measurements. Therefore, it is methodologically necessary to evaluate the validity, reliability, and factor structure of the instrument in this specific population, ensuring that the scale adequately measures the construct of adherence in the particular context of neuropsychiatric disorders.

## Methods

This study employed a cross-sectional psychometric validation design to evaluate the reliability and factorial structure of the MMAS-8 scale. Data were collected through direct administration by trained professionals during follow-up consultations at the participating institutions. Confirmatory factor analysis (CFA) was conducted to assess the structural validity of the scale, and internal consistency was evaluated using Cronbach’s alpha and McDonald’s omega. Univariate and multivariate analyses were performed using different representations of adherence. For the univariate analysis, adherence was assessed as a total score, whereas for the multivariate analysis, items were grouped into forgetting behaviors and intentional non-adherence behaviors (Table 1).

**Table 1:** Two-factor structure of the MMAS-8.

F1. Forgetting behaviors	F2. Intentional non-adherence behaviors
i1. Do you sometimes forget your medication?	i2. People sometimes skip taking their medications for reasons other than forgetfulness. Thinking back over the last two weeks, was there a day when you skipped taking your medications?
i4. When you travel or leave home, do you sometimes forget to take your medications?	i3. Have you ever reduced or stopped taking your medication without first telling your doctor because you felt worse when taking it?
i8. How often do you have difficulty remembering to take all your medications?	i6. When you feel your illness is under control, do you sometimes stop taking your medication?
	i7. Taking medication every day is a real hassle for some people. Have you ever felt frustrated about sticking to your treatment plan?

Item i5 (“Did you take your medication yesterday?”) was treated as a neutral behavioral anchor that bridges both intentional and unintentional non-adherence pathways and therefore was not classified exclusively under F1 or F2.

To ensure transparency, replicability, and methodological rigor, the study design, data collection, analysis, and reporting procedures were conducted in accordance with the Journal Article Reporting Standards (JARS) for quantitative research, as established by the American Psychological Association (Appelbaum et al., 2018).

### Participants

The sample consisted of a total of 440 Ecuadorian patients with NPD, from the cities of Guayaquil and Cuenca, Ecuador, ranging in age from 18 to 95 years (mean age = 52.83 years; SD = 21.35). The sample included 236 male and 204 female participants. All participants had previously attended psychiatric consultations. No additional inclusion or exclusion criteria were applied. No formal sample size calculation was conducted prior to data collection. However, recommendations for psychometric validation studies were taken into account, which suggest a minimum of 200 participants per factor for CFA. Since the MMAS-8 analysis was performed using a one-factor and a two-factor structure, the final sample size of 440 exceeded these recommendations, providing sufficient statistical power for the analyses conducted.

### Instrument

#### Morisky 8-item medication adherence scale (MMAS-8)

The MMAS-8 was designed by Donald E. Morisky and colleagues in 2008 as a brief and practical tool to assess patients’ adherence to medication treatment, especially in outpatient settings (Morisky et al., 2008a). The original version was conceived as a unidimensional scale measuring a single construct: medication adherence. It consists of eight items (seven dichotomous and one Likert-type), and its total score ranges from 0 to 8 points. It is classified into three levels of adherence: high (Vik et al., 2004), medium (6 to <8), and low (<6) (Morisky et al., 2008a). Although its initial design postulated a unifactorial structure, several studies have reported bifactorial or trifactorial structures, especially when analyzing populations with diabetes, hypertension, or psychiatric disorders (Lee et al., 2013; Valencia-Monsalvez et al., 2017). The psychometric properties of the MMAS-8 have been evaluated in multiple languages and contexts, showing evidence of reliability and validity in English (original), Portuguese, Arabic, Korean, and Spanish (Morisky et al., 2008a; Valencia-Monsalvez et al., 2017). The 8-item Morisky Medication Adherence Scale (MMAS-8) was used as a self-reported measure of medication adherence. This scale has been applied across different clinical settings and has shown evidence of reliability and validity; for example, Lee et al. (2013) reported adequate psychometric properties in patients with type 2 diabetes mellitus in Korea. In the present study, its psychometric properties were evaluated in an Ecuadorian sample with neuropsychiatric disorders. Therefore, it is recommended as the reference version for Spanish-language studies. However, other versions, such as the one used in patients with type 2 diabetes in Spain, have shown low internal consistency (Jöreskog and Sörbom, 1998), or which suggests that the reliability of the MMAS-8 may vary depending on the specific population and context.

### Procedure

The study was conducted during follow-up consultations at the participating institutions, with information provided through brief talks in the waiting rooms of outpatient psychiatry services between February 10 and May 20, 2024. The informational talks included a brief description of the study objectives and details regarding the voluntary nature of participation. Upon entering the consultation room, participants received an informed consent form describing the objectives, procedures, confidentiality measures, and their right to withdraw from the study at any time without consequences. Only participants who provided informed consent were administered the scale and the questionnaire.

Participants were able to complete the questionnaires at their convenience. To ensure privacy, no identifiable information was collected. All information was securely stored in a database accessible only to the principal investigator and complied with data protection regulations to safeguard participants’ confidentiality and anonymity. For this study, the Spanish adaptation developed by De las Cuevas and Peñate (2015b) for psychiatric outpatients was used.

### Analysis

The psychometric properties of the MMAS-8 were examined in several stages. First, internal consistency was assessed to determine the reliability of the scale using Cronbach’s alpha (*α*) and McDonald’s omega (*ω*). Reliability was interpreted according to standard guidelines: values above 0.70 were considered acceptable, values above 0.80 were considered good, and values above 0.90 were considered excellent (Table 2).

**Table 2:** Descriptive data of the MMAS-8 items.

	Median	Mean	SD	Skewness	SE of skewness	Kurtosis	SE of kurtosis	Shapiro–Wilk	*p*-value of Shapiro–Wilk	Min	Max
i1	1.00	0.55	0.50	−0.22	0.12	−1.96	0.23	0.63	< 0.01	0	1
i2	1.00	0.54	0.50	−0.16	0.12	−1.98	0.23	0.63	< 0.01	0	1
i3	1.00	0.55	0.50	−0.22	0.12	−1.96	0.23	0.63	< 0.01	0	1
i4	1.00	0.68	0.47	−0.77	0.12	−1.41	0.23	0.59	< 0.01	0	1
i5	1.00	0.70	0.46	−0.86	0.12	−1.26	0.23	0.58	< 0.01	0	1
i6	1.00	0.64	0.48	−0.59	0.12	−1.66	0.23	0.61	< 0.01	0	1
i7	1.00	0.60	0.50	−0.34	0.12	−1.74	0.23	0.64	< 0.01	0	2
i8	0.75	0.75	0.28	−1.13	0.12	0.63	0.23	0.80	< 0.01	0	1

The factorial structure of the MMAS-8 was evaluated using CFA to examine the original unidimensional model. The analysis employed the diagonally weighted least squares (DWLS) estimator, appropriate for ordinal data. Model fit was evaluated using multiple fit indices, including the chi-square statistic (*χ*^2^) (Steiger, 1990), the root mean square error of approximation (RMSEA) (Bentler, 1990), the standardized root mean square residual (SRMR) (Tucker and Lewis, 1973), and incremental fit indices such as the Tucker–Lewis Index (TLI) (Hu and Bentler, 1999) and the Comparative Fit Index (CFI) (Vu et al., 2025).

Following conventional guidelines, acceptable model fit was defined as RMSEA values below 0.08 and good fit as values below 0.05, SRMR values below 0.08, and TLI and CFI values above 0.90 for acceptable fit and above 0.95 for good fit. Additionally, items were retained if their standardized factor loadings exceeded 0.30 (Vu et al., 2025). Latent factor loadings were examined to assess model adequacy. Item i7 was polytomous (0–2) and showed variability comparable to the remaining items (M = 0.60, SD = 0.50).

## Results

### Participants

The sample consisted of 440 participants, with a slight predominance of men: 236 (53.64%) compared to 204 women (46.36%). Age ranged from 18 to 95 years, with a median of 55.00 and a mean of 52.83 years, indicating that most participants were in middle or late adulthood. Age variability was wide (SD = 21.35). Skewness was nearly null (0.04), and kurtosis was negative (−1.21), indicating a flattened shape relative to the normal distribution. The Shapiro–Wilk test (W = 0.95, *p <* 0.01) showed that the distribution deviated from normality, suggesting that strictly parametric tests may not have been the most appropriate. Regarding primary diagnoses (ICD-10), the most frequent group corresponded to F20–F29 (25.45%), followed by G20–G26 (22.73%) and F40–F48 (18.41%). Additional patients were classified under F00–F09 (10.23%), F30–F39 (11.14%), and G40–G47 (5.68%), while F10–F19 (2.27%), F70–F79 (2.50%), and F50–F59 (1.36%) were less common. Finally, diagnoses within F60–F69 (0.23%) presented minimal proportions. Overall, disorders in the F series accounted for 71.59% of cases, while those in the G series accounted for 28.41%, suggesting a predominantly psychiatric clinical burden in the cohort.

Regarding central tendency, most dichotomous items (i1–i7) showed a median of 1.00 and means between 0.54 and 0.70. The lowest values were observed for i2 (M = 0.54) and i1/i3 (M = 0.55), whereas the highest corresponded to i5 (M = 0.70), i4 (M = 0.68), and i6 (M = 0.64). Item i8, with a continuous/bounded ordinal response, showed a median of 0.75 and a mean of 0.75, indicating a clear concentration of high values.

Dispersion was moderate for dichotomous items (SD ≈ 0.46–0.50) and lower for i8 (SD = 0.28), indicating more homogeneous responses for the latter. Item i7 included three categories, with a mean of 0.60 and an SD of 0.50, showing variability comparable to the remaining items despite being polytomous.

Regarding distribution shape, skewness was negative for all items (between −0.16 and −1.13), suggesting clustering of responses in the upper category (a possible ceiling effect). Kurtosis was markedly negative for i1–i7 (between −1.26 and −1.98), indicating flattened distributions relative to normality; however, item i8 showed positive kurtosis (0.63), consistent with greater concentration around higher values and slightly heavier tails.

The Shapiro–Wilk normality test was significant for all items (W = 0.58–0.80; *p <* 0.01), thus rejecting normality at the item level, as expected given the dichotomous/ordinal nature of most variables. Consequently, comparisons and models involving these items benefited from categorical-data approaches: tetrachoric/polychoric correlations, robust estimators for ordinal variables (e.g., DWLS/WLSMV), and reliability coefficients suited for categorical data (KR-20 or ordinal alpha/omega).

Finally, ranges confirmed the coding: all items ranged from 0 to 1, except i7 (0–2). For joint analysis (e.g., CFA or IRT), it was appropriate to maintain i7 as a three-category ordinal item or, if comparability with the remaining items was desired, consider explicit recoding. Substantive interpretation (higher/lower adherence) depended on the coding direction of each item, although the bias toward higher values suggested responses tended toward the upper category.

### Confirmatory factor analysis (CFA) of the MMAS-8

The data were suitable for CFA. The overall KMO measure was 0.77 (item range = 0.66–0.82), indicating sufficient intercorrelation among items, and Bartlett’s test of sphericity was significant [*χ*^2^(28) = 506.92, *p <* 0.01], supporting the modeling of a latent structure.

The unidimensional model (see Table 3) showed acceptable, though not excellent, overall fit. The chi-square test for the factor model was significant [*χ*^2^(20) = 76.90, *p <* 0.01], which is expected with large N values. However, incremental indices indicated good fit: CFI/IFI/RNI = 0.92 and NFI/TLI/NNFI = 0.90. Absolute error indices were consistent with reasonable fit: RMSEA = 0.08 (90% CI: 0.06–0.10) and SRMR = 0.07. PNFI = 0.64 reflected moderate parsimony. Hoelter values suggested that with samples of ≈179–214, the model would have remained stable at *α* 0.05–0.01. With the available sample, the ability to detect model misfit was high.

**Table 3:** One-factor model.

Indicator	Std. estimate	SE	*z*-value	*p*	*Χ*^2^/df	*p*	CFI	TLI/NNFI/NFI	*ω*	*α*
i1	0.35	0.04	9.58	< 0.01	76.90/20	< 0.01	0.92	0.90	0.71	0.70
i2	0.57	0.04	14.50	< 0.01						
i3	0.54	0.04	13.97	< 0.01						
i4	0.58	0.04	13.76	< 0.01						
i5	0.47	0.04	11.86	< 0.01						
i6	0.61	0.04	14.60	< 0.01						
i7	0.38	0.04	10.20	< 0.01						
i8	0.29	0.04	7.55	< 0.01						

(RMSEA = 0.08, SRMR = 0.07), Hoelter’s Critical *N*: 178.68 (*α* = 0.05) y *N* = 213.50 (*α* = 0.01), ECVI = 0.25. Factor loadings, model fit, and reliability of the MMAS-8.

All standardized loadings were significant (*p <* 0.01) and ranged from low to moderate: i6 = 0.61, i4 = 0.58, i2 = 0.57, i3 = 0.54, i5 = 0.47, i7 = 0.38, i1 = 0.35, and i8 = 0.29. Accordingly, communalities (R^2^) were modest (mean ≈ 0.24): items i6 (0.38), i2 and i4 (0.33) explained the greatest variance, whereas i8 (0.08), i1 (0.12), and i7 (0.14) contributed the least. Residual variances were high for i8 (0.92), i1 (0.88), and i7 (0.86), suggesting substantial item-specific variance not captured by the general factor (e.g., memory/executive difficulties or treatment-related burden). Internal consistency was acceptable for research use (*ω* = 0.71; *α* = 0.70). Overall, global fit indices were consistent with an acceptable unidimensional solution for the MMAS-8 in this sample (good CFI/IFI/RNI; acceptable RMSEA/SRMR), although the weaker items warrant cautious interpretation.

The results of the bifactorial analysis of the MMAS-8 questionnaire are presented in Table 4, distinguishing two main dimensions: forgetting behaviors and intentional nonadherence behaviors. In the first factor, items i1, i2, i4, i5, and i8 showed factor loadings between 0.30 and 0.59, all statistically significant (*p <* 0.01). These results indicate that the items are adequately associated with the forgetting construct, with item i8 standing out for its lower loading (0.30), reflecting weaker association with the dimension. In the second factor, items i3, i6, and i7 showed moderate-to-high loadings, ranging from 0.39 to 0.65, also significant (*p <* 0.01), demonstrating that they consistently represent the dimension of intentional nonadherence.

**Table 4:** Two-factor model.

Factor	Indicator	Std. estimate	SE	z-value	*p*	*Χ*^2^/df	*p*	CFI	TLI/NNFI/NFI	*ω*	*α*	HTMT
Factor 1	i1	0.36	0.04	9.67	< 0.01	76.31/19	< 0.01	0.92	0.90	0.72	0.70	0.89
	i2	0.59	0.04	14.17	< 0.01							
	i4	0.59	0.04	13.38	< 0.01							
	i5	0.48	0.04	11.60	< 0.01							
	i8	0.30	0.04	7.58	< 0.01							
Factor 2	i3	0.57	0.04	13.10	< 0.01							
	i6	0.65	0.05	13.29	< 0.01							
	i7	0.39	0.04	9.90	< 0.01							

Factor 1 = Forgetfulness behaviors y Factor 2 = Intentional non-adherence behaviors (RMSEA = 0.08, SRMR = 0.07), Hoelter’s Critical *N*: 177.45 (*α* = 0.05) y *N* = 212.85 (*α* = 0.01), ECVI = 0.25. Factor loadings, model fit, and reliability of the MMAS-8.

Regarding model fit, the *χ*^2^/df statistic (76.31/19, *p <* 0.01) was significant, suggesting a discrepancy between the model and the data. However, given the sensitivity of this index to sample size, complementary indicators are more relevant. In this case, CFI = 0.92 and TLI = 0.90 met acceptance criteria (>0.90), indicating that the bifactorial model provided adequate fit.

Instrument reliability was further supported by satisfactory values. Omega (*ω* = 0.72) and Cronbach’s alpha (*α* = 0.70) were within acceptable ranges, reflecting adequate internal consistency. Additionally, the HTMT index = 0.89 supported discriminant validity between the two factors, as it was just below the critical threshold of 0.90, confirming adequate differentiation between forgetting and intentional nonadherence.

Overall, findings support the validity of the bifactorial model for the MMAS-8, demonstrating that the instrument allows differentiated assessment of unintentional forgetting behaviors and intentional nonadherence behaviors. The results reflect adequate fit and consistent reliability, although attention should be given to items with lower loadings, such as i8, which may require review in future studies to improve model precision.

## Discussion

### Unifactorial studies

The findings of this study support the usefulness of the MMAS-8 for assessing medication adherence in patients with NPD. Its internal consistency and factor analysis results indicate a solid structure in both unidimensional and multidimensional models, consistent with the observations of De las Cuevas and Peñate (2015b), who reported acceptable reliability and good diagnostic discrimination in psychiatric outpatients.

However, Nguyen et al. (2014), emphasize, in their systematic review, the need for cautious interpretation of MMAS-8 results due to methodological limitations in original validations, including absence of test–retest analysis and unclear specification of which adherence components are captured. This caution is particularly important in NPD, where cognitive impairment, poor insight, or intentional refusal may influence responses. Thus, despite supporting internal validity, MMAS-8 use should be complemented with additional clinical tools.

Furthermore, findings by Vu et al. (2025), in patients with bipolar disorder show that factors such as male sex, low education, polypharmacy, and limited family support are associated with lower adherence scores. These results reinforce the criterion validity of the MMAS-8 and highlight the importance of family support as a protective factor, suggesting that family-centered interventions may improve adherence and treatment outcomes in chronic mental disorders.

The study by Çoban et al. (2025), supports the convergent validity of the MMAS-8 by showing a significant positive correlation between adherence and quality of life and functioning, indicating that higher adherence is linked to better psychosocial adjustment and well-being. This reinforces the clinical relevance of the MMAS-8, suggesting that it relates not only to adherence behavior but also to broader clinical outcomes.

Medication adherence in neuropsychiatric disorders is influenced by numerous clinical, psychological, social, and contextual variables. Research highlights that factors such as predominant polarity in bipolar disorder, impulsivity, insight, symptom severity, and quality of life significantly affect adherence, emphasizing the need for instruments sensitive to these dimensions (Borrás-Gallén et al., 2025). Although the MMAS-8 is widely used, its psychometric properties have been questioned across different clinical populations.

The study by Martínez-Pérez et al. (2021b) in patients with type 2 diabetes revealed low internal consistency (*α* = 0.40) and a three-factor structure, suggesting that the scale does not measure a single construct. Although it showed good temporal reliability, its structural limitations indicate that the MMAS-8 may not be appropriate without adaptations in populations with specific cognitive characteristics, as seen in NPD. Similar findings were reported by Borrás-Gallén et al. (2025), who evaluated the scale in patients receiving oral anticoagulants, observing moderate internal consistency (Omega ≈ 0.69), low temporal stability, and the absence of a clear unidimensional structure. These results reinforce the idea that the MMAS-8 requires population-specific validations before being used as a reliable measure of adherence.

Nevertheless, the scale has shown promising results in psychiatric contexts. The study by De las Cuevas and Peñate (2015b), conducted with outpatient psychiatric patients in Spain, found a clear unidimensional structure and good concurrent validity with relevant psychological variables such as locus of control and attitudes toward medication. Although that study did not report internal reliability or temporal stability, its findings suggest that the MMAS-8 can capture important aspects of adherence in mental health populations. However, these results may not be fully transferable to Ecuador, where differences in health-system organization (e.g., access to medications and continuity of follow-up) and sociocultural/linguistic features (e.g., health literacy, stigma, and everyday language use) could influence item interpretation and response patterns. Accordingly, our study empirically evaluated whether the MMAS-8 retains adequate reliability and factorial structure in Ecuadorian patients with neuropsychiatric disorders.

Additionally, recent studies reinforce the importance of considering contextual and sociodemographic variables. For example, Vu et al. (2025) found in Vietnamese patients with bipolar disorder that non-adherence is associated with being male, having a low educational level, and receiving complex treatments, while family support and marital status (being married) are associated with better adherence. These data demonstrate the substantial weight of social factors, consistent with the findings of Zewdu et al. (2025), whose meta-analysis linked non-adherence with self-stigma, lack of insight, negative attitudes toward treatment, and lack of social support. These studies confirm that adherence in mental disorders does not depend solely on the patient or the treatment, but also on the social environment and the healthcare system.

Furthermore, evidence also supports a direct relationship between adherence, quality of life, and functioning. A cross-sectional study in outpatients with bipolar disorder showed that higher adherence levels, measured by the MMAS-8, were significantly associated with better quality-of-life scores (QoL. BD) and functional levels (BDFQ), suggesting that promoting adherence has positive effects on overall well-being (Borrás-Gallén et al., 2025). This correlation supports the use of the MMAS-8 not only as an assessment tool but also as an indirect marker of relevant clinical outcomes.

Taken together, these findings reveal a dual reality regarding the MMAS-8: on one hand, its potential usefulness in capturing psychological and clinical aspects associated with adherence in psychiatric contexts; on the other, its psychometric limitations when applied to populations with diverse clinical conditions, such as somatic diseases or patients with complex therapeutic regimens. The variability in its psychometric performance indicates that it cannot be considered a universal tool without contextual adjustments.

Therefore, validation of the MMAS-8 in patients with NPD must rigorously consider variables such as cognitive impairment, insight, symptom severity, and psychosocial environment to ensure that items are interpreted appropriately and that the results accurately reflect the adherence construct. Moreover, given the influence of cultural, social, and clinical factors, complementary use of other instruments or strategies is recommended to enable a more holistic assessment, along with the development of personalized interventions aimed at improving adherence through multiple avenues—educational, familial, therapeutic, and community-based.

Medication adherence is a crucial factor in the management of chronic diseases, especially in patients with both medical and psychiatric comorbidities, such as type 2 diabetes and depressive disorders. In this context, multiple studies in Mexico have shown a consistent relationship between depressive symptoms and non-adherence, highlighting the negative influence of mental health on metabolic control and overall well-being. Studies such as those by Mora-Romo (2022), López Domínguez (2024), Sánchez-Cruz et al. (2016), and Bastida-Reyes et al. (2023) conclude that depression and stress negatively affect adherence to both pharmacological and nutritional treatment, which are essential components of diabetes management.

These findings are consistent with those reported by Medina-Rodríguez and Lugo-Bautista (2024), who validated the MMAS-8 in a sample of Mexican patients with depressive disorders and type 2 diabetes. The scale demonstrated high internal consistency (*α* ≈ 0.88), supporting its use as a reliable tool for measuring adherence in this complex population. The fact that the MMAS-8 performs adequately in patients with simultaneous medical and psychiatric conditions reinforces its value in clinical contexts where barriers to therapeutic follow-up are multiple and overlapping.

Consistent with this evidence, the study by Montesinos and Tuesta (2022) in Peru adapted and validated the MMAS-8 in patients with psychiatric diagnoses receiving teleconsultation—a modality increasingly common in settings with limited in-person access. The results confirmed that the scale is valid and reliable in this context, highlighting its ability to identify patients at risk of treatment dropout or non-compliance. This is especially relevant considering that adherence in mental health varies widely, with reported rates between 20 and 72% across studies.

The added value of this study lies in demonstrating that the MMAS-8 remains useful even in virtual environments, provided that adequate linguistic and cultural adaptation is ensured. Nevertheless, the authors emphasize the need for future research linking adherence—as measured by this scale—with objective clinical outcomes, and exploring the role of sociodemographic and contextual variables in adherence during remote care.

Furthermore, studies from other Latin American countries have reinforced the potential usefulness of the MMAS-8 in various clinical conditions. In Colombia (Montesinos and Tuesta, 2022), validation of an abbreviated five-item version in hypertensive patients showed acceptable reliability (Omega = 0.71; Spearman = 0.89), suggesting that even modified versions of the scale can be useful when adjusted to the characteristics of the target population. In Chile (Berlowitz et al., 2017), its application in older hypertensive adults showed high sensitivity (86%) but moderate specificity (56%), positioning the MMAS-8 as an effective tool for early detection of non-adherence—though it should be complemented with other methods to avoid false positives.

Despite these positive findings, Ecuador lacks specific studies validating the use of the MMAS-8 in its population, limiting the generalizability of results obtained in other countries. Although data from Peru, Colombia, Chile, and Mexico provide encouraging evidence regarding the scale’s validity and reliability in Latin American contexts, it is essential to conduct a local validation ensuring linguistic and cultural adequacy. Such adaptation would allow for accurate assessment of adherence in Ecuadorian patients and facilitate the development of personalized interventions based on valid data.

### Multifactorial studies

The MMAS-8 does not have a single universally accepted factorial structure, as it depends on the population and the psychometric analysis applied. In general, its structure was originally designed as a unidimensional scale to measure medication adherence. That is, it was initially conceived with one factor. According to psychometric studies, some factor analyses confirm a single factor that explains most of the variance (adherence), while other studies have identified two-factor solutions, typically distinguishing between forgetting behaviors (items related to memory lapses and oversight) and intentional non-adherence behaviors (when the person decides not to take the medication). Thus, although the scale is theoretically conceived as unidimensional, it may empirically show one or two factors depending on the analysis and the population studied.

In this study, a two-factor analysis was also performed. Several studies have evaluated the factorial structure of the MMAS-8 across different populations and clinical contexts, showing evidence that the scale may present a multidimensional structure depending on the sample’s profile. For example, Zongo et al. (2016), identified two factors in patients with type 2 diabetes: one related to intentional non-adherence and the other to unintentional non-adherence, suggesting a possible behavioral differentiation in adherence measurement (Cabral et al., 2018). Similarly, Song et al. (2013), in the validation of the Portuguese version, supported a bifactorial model distinguishing between voluntary and involuntary components of non-adherence.

On the other hand, studies in Asian populations have also revealed multidimensional structures. In Korea, Lee et al. (2013) reported three factors explaining more than 60% of the variance in patients with type 2 diabetes. In rural older adults with hypertension, another Korean study identified two dimensions with acceptable internal consistency (Martínez-Pérez et al., 2021b).

Outside Asia, a Spanish study in patients with type 2 diabetes found that the exploratory factor analysis of the MMAS-8 yielded a three-component structure, suggesting that medication adherence may involve differentiated dimensions in this population (Shin and Kim, 2013). In contrast, in psychiatric outpatients, De las Cuevas and Peñate (2015b) reported a clear tendency toward a one-factor solution, although item 7 did not clearly fit within that structure and model fit improved when this item was excluded.

Taken together, these findings suggest that although the scale was originally designed as unidimensional, its psychometric performance varies across cultural and clinical contexts. This reinforces the need to conduct population-specific factor analyses before applying the scale in new groups.

Other tools besides scales can be used to measure adherence in neuropsychiatric disorders such as depression. For example, a study by Abdul Ghafur et al. (2024), which reviewed 15 studies (2013–2023) to evaluate the methods used to determine antidepressant adherence in adults with depression, revealed a wide variety of tools, highlighting self-report measures as the most practical in primary care and psychiatry. Scales such as the MARS showed acceptable reliability and validity, while others such as the MAQ, BARS, MMAS, MGLA, BMQ, and DAI-10 showed good validity or reliability. Objective methods such as pharmacy refill data, pill counts, and serum levels were also identified. However, no single method demonstrated sufficient consistency across all phases of adherence, so the authors recommend a combined approach for a more accurate assessment (Martínez-Pérez et al., 2021a).

In a systematic review by Chong et al. (2011) on interventions to improve antidepressant adherence in patients with unipolar depression, the authors found that 57% of interventions achieved significant improvements in adherence, while 43% showed positive effects on both adherence and clinical outcomes for depression. The most effective interventions were multifaceted, characterized by combined strategies such as patient education, telephone follow-up, treatment support, and feedback to primary care physicians, as well as the active involvement of mental health specialists and proactive care management. In contrast, purely educational interventions did not demonstrate significant effectiveness, suggesting that improving adherence requires complex and comprehensive behavioral approaches.

### Limitations

The study has several limitations: the sample was restricted to NPD patients from two Ecuadorian cities, limiting generalizability. More diverse samples are needed to assess the MMAS-8’s cross-cultural validity and to examine its discriminant validity against other adherence measures. No prior power analysis was performed, although the final sample size meets recommended standards for psychometric validation studies.

We acknowledge that the study population is heterogeneous and that different neuropsychiatric disorders, such as Parkinson’s disease, Alzheimer’s disease, and epilepsy, present distinct clinical, cognitive, and therapeutic profiles that could influence adherence patterns. However, the study’s objective was not to compare specific behaviors across diagnoses, but rather to evaluate the scale’s psychometric performance in a real-world clinical setting that reflects the typical diversity of specialized services. Validation focused on determining whether the instrument maintains internal consistency, factor structure, and measurement stability in a large and clinically representative sample. Additionally, stratified/exploratory analyses were conducted to examine the stability of results across diagnostic subgroups, allowing for the identification of potential variations without compromising the instrument’s overall applicability. Therefore, our findings should be interpreted with caution and are limited to populations with neuropsychiatric disorders as a whole, recognizing that future studies with larger, specific samples for each diagnosis could explore particular differences in greater depth.

In this study, a formal statistical power analysis was not performed because the design was observational, descriptive, and exploratory, based on a non-probability sample comprised of all patients available during the data collection period. The research did not aim to test specific hypotheses between groups with equivalent sample sizes, but rather to estimate adherence levels under real-world clinical conditions and explore trends in different neuropsychiatric diseases, whose sample sizes naturally differ according to their prevalence and healthcare demand. In this context, calculating power *a priori* loses methodological relevance, since a minimum expected effect size was not defined, nor was a uniform sample size planned for each group. Furthermore, the inclusion of all eligible cases reduces selection bias and strengthens the ecological validity of the study. However, it is acknowledged that unequal sample sizes may limit the precision of some inferential comparisons, which is noted as a limitation. Instead, the presentation of effect sizes and confidence intervals is prioritized for a more informative interpretation of the results.

### Final reflections

The study found that participants were mainly adults with psychiatric conditions, underscoring the need to assess medication adherence in this group. MMAS-8 items showed ceiling effects and non-normal distributions, supporting the use of categorical-data analyses. A one-factor model demonstrated acceptable fit, though items i1, i7, and i8 contributed weakly. A two-factor model, separating forgetting from intentional non-adherence, showed comparable or slightly better fit and good discriminant validity. Overall, the MMAS-8 proved valid and reliable, with the two-factor structure offering a more accurate representation of adherence, though some items may need revision.

## Data Availability

The original contributions presented in the study are included in the article/Supplementary material, further inquiries can be directed to the corresponding author.
